# The Slavery of the *h-index*—Measuring the Unmeasurable

**DOI:** 10.3389/fnhum.2016.00556

**Published:** 2016-11-02

**Authors:** Grzegorz Kreiner

**Affiliations:** Department of Brain Biochemistry, Institute of Pharmacology, Polish Academy of SciencesKrakow, Poland

**Keywords:** *h-index*, bibliometrics, citations, nobel prize, research evaluation

## Introduction

Last year we “celebrated” the 10th anniversary of the invention of the *h-index* (also known as the *Hirsch factor*; Hirsch, 2005), an indicator created by Jorge E. Hirsch, that attempts to measure the achievements of a research scientist. However, it not only appears that *h*-*index* has taken on a life of its own but also that the popularity of this formula currently surpasses the initial idea for its use envisioned by the inventor. Originally introduced as a simple characterization of the scientific output of a researcher (Hirsch, 2005), the *h-index* has come to be uncritically regarded as a “magic tool” that is applied to measure what is unmeasurable—the quality of science. As a result, it has become a “must have” indicator when applying for funds or a new position. Surprisingly, many decision-makers apparently are not fully aware of what it represents. According to its inventor, the *h-index* is the number of papers coauthored by the investigator with at least *h* citations each (Hirsch, 2005). That is, to be the proud owner of a high *h-index*, it is not sufficient to have authored many articles or for some of them to have been extensively cited. Rather, such an achievement must satisfy both issues: a considerable number of articles must be highly cited, which is reflected by ranking them according to the number of citations they have collected, and finding the one where the position on the list equals or is less than the number of the citations it garnered.

### The pros and cons of the *h-index*

The initial idea of Hirsch was to discriminate the investigators who are persistently productive from those who experienced an isolated auspicious moment in their scientific life, and who currently only cut coupons from their popularity roll. Nevertheless, it assumes that researcher A, who published a breakthrough story that was extensively cited, should deserve less respect than researcher B who publishes often and regularly; however, the outcome of the latter's work has not contributed yet to any remarkable discovery. A good example is the inventor of the RNA isolation method, Piotr Chomczynski. He has in total over 65 000 citations to which he contributed almost exclusively (92.9% of all citations) with one single paper regarding the method he introduced in 1987 (Chomczynski and Sacchi, 1987). His current *h-index* is 23, relatively low for such a prodigious number of citations. Yet, could we imagine working with RNA over the past number of years without possessing this simple technique that is now the principle of virtually every commercial protocol related to RNA extraction? Notwithstanding, it might be argued—depreciating that discovery—that because this method is so simple, someone else would have been discovered it sooner or later. In response, let me cite my former mentor, professor Günther Schütz, who would disprove similar arguments with a simple statement: *If you say this is so trivial, why was it not you who discovered PCR?* Breakthrough discoveries are not solely dependent on sophisticated science.

Another problem with the *h-index* is the impossibility of comparing the investigators during different stages of their careers (even assuming comparisons among those representing the same field, which is another ambiguous factor). There is a certain correlation between the age of an investigator and *h-index*. Clearly and in any case, some of articles will accumulate citations and this number will increase over the time since they were first published. However, even the comparison between investigators at a similar career stage may often be misleading, particularly among young post-docs whose careers have just begun. We must be honest and acknowledge the fact that it is a rare occurrence that right after completing a PhD, such an investigator is able to independently decide about own career development. Mostly, the scientific achievements at this stage are primarily a derivative of the power of the PhD mentor and the reputation of the hosting institution.

Another issue contributing to *h-index* limitations is that many research groups have different regulations regarding authorship. It is assumed that a researcher's name will be added to the authorship list only after considerable contribution has been made to the published work. However, what occurs fairly often is that being a “middle man” on the listing does not necessary reflect the significant contribution and, worth to be emphasized, the *h-index* does not differentiate between article authors who hold the most valuable first and last authorship position and those wherein the author's name appears as one among perhaps even 100 authors, as occurs with articles containing vast meta-analyses of clinical data.

Although, some of these concerns were raised in the discussion part of the initial paper published by Hirsch (2005), they were overshadowed by the enormous popularity of this tool and its indiscriminate application. Despite of many critical yet unofficial discussions about the *h-index*, its limitations, and perhaps dangerous influence on science, the topic—if only tackled by publications—is usually narrowed to the pursuit of more and more sophisticated bibliometrics and proposals of new indicators (Bharathi, 2013; Biswal, 2013; Díaz et al., 2016; Würtz and Schmidt, 2016). The critical voices seem to be less represented, nevertheless there are of course existing articles pointing out that past achievements of the scientists may not necessarily be correlated with future success and that all such rankings need context which means that the best method to gain an impression of the quality is still simply reading the papers (Wendl, 2007; von Bohlen Und Halbach, 2011). Perhaps the combination of looking into the context of the particular paper and the journal reputation gives the best assumption in this matter, however even this approach can be biased toward personal preferences and requires considerable amount of time.

Last but not least, when examining the reasons why the *h-index* is not trustworthy, we should not neglect issues regarding fraud. Because the *h-index* does not discriminate self-citations, it is not difficult to predict that even the investigators who are poorly cited by others but who publish prodigiously, citing mostly themselves, will easily increase their *h-index* in the long run. Moreover, some evidence of misuse has been reported involving artificially pumped *h-indices* based on i.e., regular cross-citations between good friends (Kotov, 2010).

### The *h-indices* of nobel prize winners

**Figure 1:**
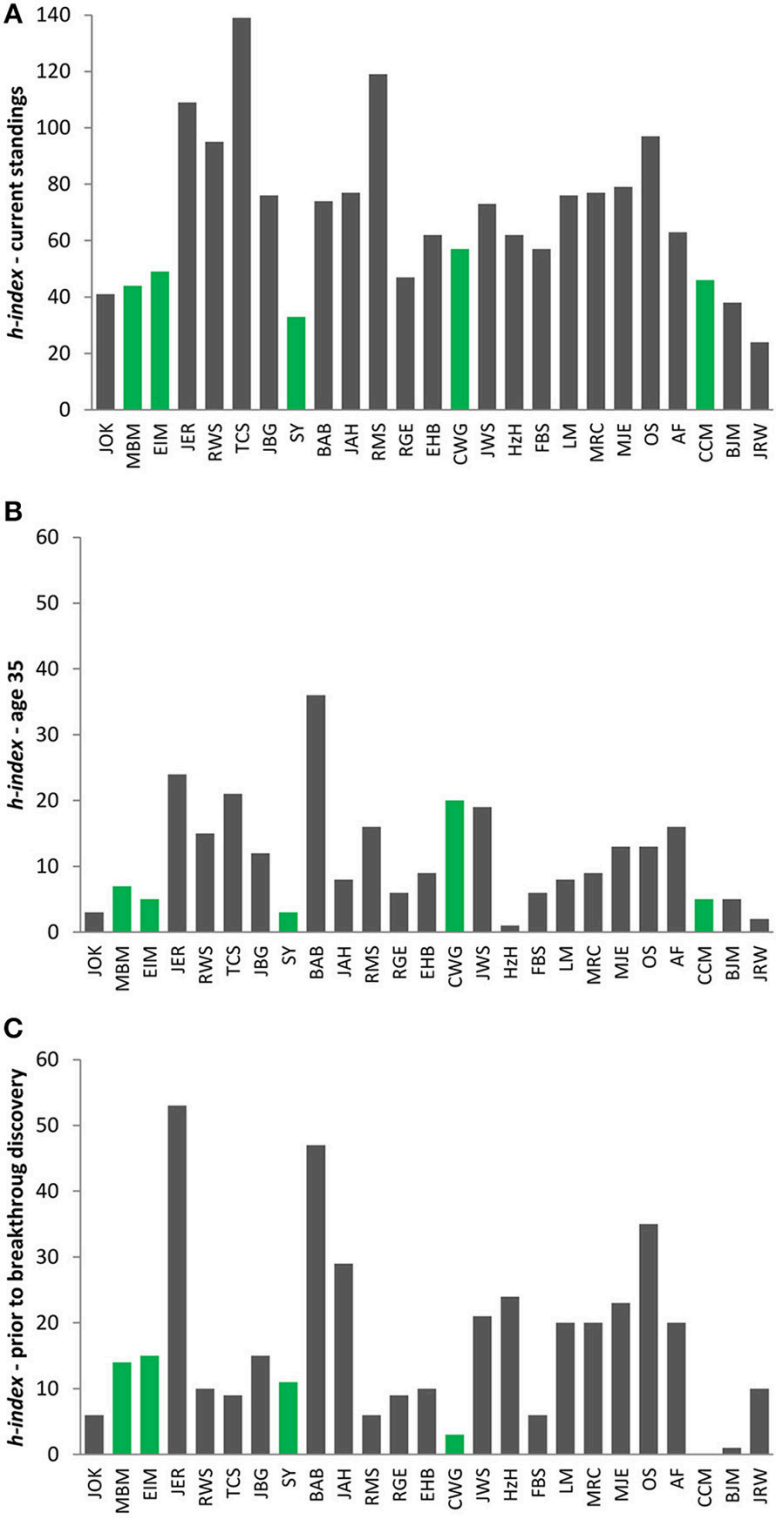
**The ***h-indices*** of Nobel Prize laureates granted during the period 2005–2015; (A) current standings; (B) ***h-indices*** at the age of 35 years; (C) ***h-indices*** for the year prior to the breakthrough discovery**. Green bars, Nobel Prize laureates born after 1960. Following papers were selected as the firstly announced breakthrough discovery of evaluated laureates: John O'Keefe, JOK (Morris et al., 1982); May-Britt Moser, MBM (Fyhn et al., 2004); Edvard I. Moser, EIM (Fyhn et al., 2004); James E. Rothman, JER (Söllner et al., 1993); Randy W. Schekman, RWS (Novick et al., 1980); Thomas C. Südhof, TCS (Südhof et al., 1987); John B. Gurdon, JBG (Gurdon et al., 1971); Shinya Yamanaka, SY (Takahashi and Yamanaka, 2006); Bruce A. Beutler, BAB (Poltorak et al., 1998); Jules A. Hoffmann, JAH (Lemaitre et al., 1996); Ralph M. Steinman, RMS (Steinman and Cohn, 1973); Robert G. Edwards, RGE (Edwards, 1965); Elizabeth H. Blackburn, EHB (Greider and Blackburn, 1985); Carol W. Greider, CWG (Harley et al., 1990); Jack W. Szostak, JWS (Lundblad and Szostak, 1989); Harald zur Hausen, HzH (Devilliers et al., 1987); Françoise Barré-Sinoussi, FBS (Barré-Sinoussi et al., 1983); Luc Montagnier, LM (Barré-Sinoussi et al., 1983); Mario R. Capecchi, MRC (Thomas and Capecchi, 1987); Sir Martin J. Evans, MJE (Evans and Kaufman, 1981); Oliver Smithies, OS (Blattner et al., 1977); Andrew Fire, AF (Fire et al., 1998); Craig C. Mello, CCM (Mello et al., 1991); Barry J. Marshall, BJM (Marshall and Warren, 1984); J. Robin Warren, JRW (Marshall and Warren, 1984). The *h-indices* of the examples provided were calculated according to the Web of Science Core Collection database (Thomson Reuters).

Great discoveries require time for experiments. Furthermore, quality investigational pursuits require time for planning, critical thought, discussion, repetition. This process cannot be conducted in haste and under pressure to simply publish all that has been collected. Isaak Newton was said to think twice and to examine all of his discoveries time after time until he finally decided to publish them. Currently, with such an approach, he would most likely have been released nowadays from the university due to the lack of progress in his research. Seemingly, this example perhaps would be difficult to prove. However, consider instead scenarios that are closer to reality—the Nobel Prize laureates in Physiology & Medicine, an unbiased selection of preeminent scientists in life sciences. Do they actually possess *h-indices* above 100 or thereabouts? Indeed and not surprisingly, many of them do. However, there are also laureates who would have inevitably encountered roadblocks had their *h-index* been the decisive factor in their nomination. If we consider the last 25 laureates from the period of the recent 10 years, the outcomes are somewhat surprising. The *h-index* values vary among these esteemed professors within the range of 24–139 (Figure 1A), although no one would rightfully propose that one of these laureates should be regarded as a 5-fold better scientist than another. Clearly, this may again have reflected the time when the Nobel-worthy discovery was made and simply the age of the laureate, another problematic issue raised in one of recent commentaries in *Nature* journal (Fortunato, 2014). Nonetheless, even when comparing the laureates by their *h-indices* at the same age of 35 years, the average age of a young post-doc (Figure 1B), or the year before the laureates' famous discoveries were first announced in top-flight journals (Figure 1C), no correlation exists and moreover, examples may be found of investigators who had virtually no bibliometric track records. This is also true among relatively young Nobel laureates (those born >1960) whose careers were certainly influenced by the IT era from the very beginning (Figures 1A–C, green bars). It could be surmised that in many cases the moderate *h-indices* at this stage are simply correlated to the persistence in pursuing the hypothesis that ultimately was proven, thereby yielding a scientific breakthrough.

### A lesson from poland

We have had a unique experience in Poland, arising from the Communist period of our history, when all work efforts were required to be quantified by numbers, that were the most important, even though the outcomes of that work were far from expected. That was a time during which, e.g., a car factory would proudly announce that it had produced the 1-millionth vehicle, although it completely disregarded that it was barely possible to start the engine in most of the cars or to exit a parking spot because of a variety of mechanical failures.

It appears that looking through the lens of the *h-index*, we are perilously reverting back to those times. What is conveyed in the article, the quality of the work and the actual impact on the topic in focus does not appear to be important. What counts is quantity, not quality. With more articles and more citations, the magic “h” letter will increase. As a result, this activity will considerably increase the approval chances of grant application or will lend favor to tenure position evaluation. Yet, what will be the feedback regarding these articles? Can anything be learned from them? Apparently, this is out of the question—it does not affect the *h-index*.

Poland is now enjoying a good period in its history. As a result of maintaining the gross domestic product in the black, despite worldwide economic crises, the monies spent on research are continuously increasing. Moreover, several attempts and programs are underway that aim to reorganize science in Poland to be more competitive and efficient. However, all these efforts may backfire if we remain stuck to the easy and old-fashioned way of evaluating our work.

Unquestionably, we scientists must somehow be evaluated as our job is based on spending public monies and such work should be accomplished in an efficient manner. However, the critical evaluation of science requires a great deal of effort and cannot be done using a simple comparison of numbers. As stated by the Hirsch himself, the single number can give only the rough approximation to an individual's researcher profile with many other factors contributing as well (Hirsch, 2005). The overall assessment is also affected by an average number of citations per paper, the quality of journals where the work was published, and goes far beyond the publication track records including other scientific achievements i.e., invited lectures, international experience (post-doctoral fellowships, sabbaticals, collaborations), project managements.

Another issue is the system of evaluation built upon assumption that future success of the project depends on the previous achievements of the applicant. Thus, it remains tempting for some of the grant reviewers to assess the applicants solely through the prism of their respective *h-indices*, and such a relentless pursuit of bibliometric factors may bring global science to its knees, leaving no room for independent research that may lead to the elucidation of fundamental biological quandaries.

## Author note

The author is a research associate at the Department of Brain Biochemistry, Institute of Pharmacology, Polish Academy of Sciences (IF PAS), Krakow, Poland. A former post-doc (2005–2008) at the German Cancer Research Center (DKFZ), Heidelberg, Germany. The PI on 2 research grants funded by the Polish National Science Center (NCN).

## Author contributions

GK designed, calculated data, and wrote the paper. No other person was involved.

### Conflict of interest statement

The author declares that the research was conducted in the absence of any commercial or financial relationships that could be construed as a potential conflict of interest.
